# Identification of Phenolics Profile in Freeze-Dried Apple Peel and Their Bioactivities during In Vitro Digestion and Colonic Fermentation

**DOI:** 10.3390/ijms24021514

**Published:** 2023-01-12

**Authors:** Hafza Fasiha Zahid, Akhtar Ali, Chaminda Senaka Ranadheera, Zhongxiang Fang, Said Ajlouni

**Affiliations:** School of Agriculture and Food, Faculty of Veterinary and Agricultural Sciences, The University of Melbourne, Melbourne 3010, Australia

**Keywords:** apple peel polyphenols, LC-ESI-QTOF-MS/MS, in vitro digestion, prebiotic potential, short chain fatty acids

## Abstract

Freeze-dried apple peel powder (Fd-APP) was subjected to in vitro digestion and colonic fermentation to evaluate the variations in its phenolic composition, bioactivities (antioxidant activity, α-amylase, and α-glucosidase inhibition), and fecal metabolic outputs. A total of 88 phenolics were tentatively identified, of which 51 phenolic compounds were quantitated in Fd-APP sample extracts before digestion, and 34 were released during subsequent phases of digestion. Among these, phenolic acids showed the highest bio accessibility index (BI) of 68%, followed by flavonoids (63%) and anthocyanins (52%). The inhibitory functions of Fd-APP extract against α-amylase and α-glucosidase pre- and post-digestion were moderate and ranged from 41.88 to 44.08% and 35.23 to 41.13%, respectively. Additionally, the antioxidant activities revealed a significant (*p* ≤ 0.05) decline during the in vitro digestion. However, the colonic fermentation stage presented different products where the intact parent phenolic compounds present in Fd-APP were utilized by gut microbes and produced various phenolic metabolites such as 3- hydroxyphenyl acetic acid (3-HPAA), ferulic acid (FA), 3-(4-hydroxyphenyl) propionic acid (3,4 HPPA) and 4- hydroxybenzoic acid (4-HBA). Furthermore, colonic fermentation of Fd-APP accelerated the production of short-chain fatty acids (SCFAs), with acetic acid being the most prevalent (97.53 ± 9.09 mM). The decrease in pH of fermentation media to 4.3 significantly (*p* ≤ 0.05) enhanced counts of *Bifidobacterium* (10.27 log CFU/mL), which demonstrated the potential prebiotic effects of Fd-APP. These findings indicated that the consumption of apple peel as a constituent of novel functional foods may support and protect the intestinal microbiota and consequently promote human health.

## 1. Introduction

Apples are one of the most popular and frequently consumed fruits worldwide and are excellent reservoirs of bioactive phenolic compounds and dietary fiber. Both dietary fibers and polyphenols (PPs) present in large quantities in apple peel and flesh, however, based on a dry weight (dw), apple peel contains about 4 times more of total phenolic contents than the flesh [1]. The predominant phenolic compounds in apple peels are flavonols, plant sterols, dihydrochalcones, procyanidins, and anthocyanins, whereas hydroxycinnamic acids are more prevalent in the flesh [2]. However, the PPs contents differ markedly among various apple varieties. Previous epidemiological research on apple peels have consistently demonstrated the health advantages of polyphenolic compounds, such as the prevention of metabolic dysfunction and the regulation of gut flora [1,3,4]. Koutsos et al. [5] tested the in vitro fermentability of three whole apple varieties using fecal inoculum from human subjects and the authors reported altered microbiota and short chain fatty acids (SCFA) profile, yet the authors reported varietal differences. Nevertheless, the absorption and metabolism of phenolics during digestion, which contribute to the bioavailability of apple phenolics, are necessary for the beneficial biological effects of apple bioactive components [5,6].

Apple peels as food constituents are expected to pass through digestive system after ingestion. The stability, release, and bioavailability of PPs from apple peel are affected by certain intrinsic and extrinsic factors such as their interactions with other food components (glycosylation and esterification with dietary fibers), absorption kinetics of gastrointestinal tract, and their modification by the liver [7]. The structure and disposition of PPs in a food matrix determine their ability of quenching free radicals and inhibitory actions against digestive enzymes such as α-glucosidase and α-amylase along the gastro-intestinal tract. During gastrointestinal digestion, PPs undergo diverse structural modifications before and after their absorption in the small intestine. Small intestinal epithelial cells facilitate the absorption of PPs via active or passive pathways, where they are further transformed into their methylated, sulphated, or glucuronidated forms and then enter the systemic circulation [8]. Studies have demonstrated that a substantial proportion of apple PPs together with non-digestible fibers escape absorption in the small intestine. Consequently, PPs/non-digestible fiber remain inaccessible by the digestive enzymes, and reach the colon almost intact, where they are fermented and bio-converted by the colonic microbiota producing metabolites [9]. Numerous in vitro studies, using human fecal samples for simulated colonic fermentation have reported the conversion of indigestible food components into SCFAs by several genera of colonic bacteria, including *Eubacteria*, *Bacteroides*, and *Bifidobacteria* [3,10]. These bacteria release certain enzymes which are specific to the action of hydrolyzing complex bonds between carbohydrates and polyphenols [5]. Thus, fermentation of these dietary phenolics and fiber has been reported to improve their relative bioactivity than their precursors [11]. Moreover, polyphenols and their metabolites can modulate the composition of gut microbiota by impeding the growth of pathogens and encouraging beneficial microbes, thus acting as potential prebiotics [3].

A plethora of laboratory studies previously investigated the beneficial effects of apple components during simulated colonic fermentation processes using in vitro and in vivo models [1,3,12]. Only a limited number of studies focused on the role of apple peel as prebiotic ingredient [10]. However, to the best of our knowledge, bio accessibility studies of apple peels involving entire in vitro digestion and colonic phases are scarce. This present work provides a comprehensive report that assessed the stability and bio accessibility of PPs in freeze-dried apple peel powder (Fd-APP) using both in vitro digestion and colonic phases. To quantify their potential health benefits, the antioxidant activities, total phenolic content as well as the inhibitory activity of phenolics against α-amylase and α-glucosidase were investigated. The effects of insoluble Fd-APP on the production of SCFAs along with variations in pH and composition of fecal microbiota were also examined.

## 2. Results and Discussion

### 2.1. Total Phenolic Contents and Antioxidant Capacity of Raw and Digested Extracts of Fd-APP

**Figure 1:**
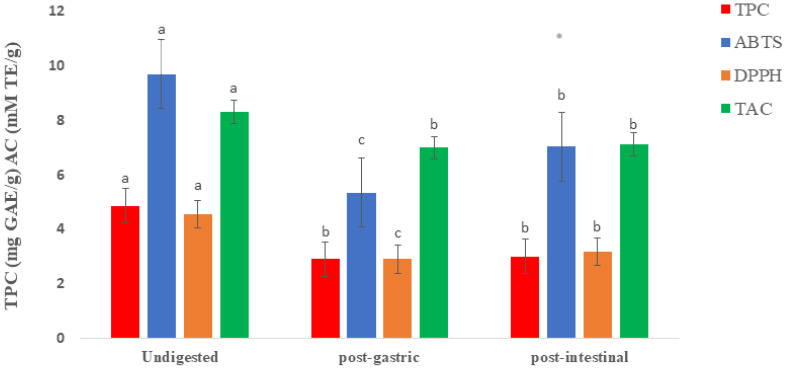
Total phenolic contents (TPC) and Antioxidant capacities using ABTS [2,2′-azino-bis (3-ethylbenzothiazoline-6-sulfonic acid], DPPH [2,2-diphenylpicrylhydrazyl], and TAC [total antioxidant capacity] methods of phenolics released from Fd-APP before and after in vitro gastrointestinal digestion. The values are means of tests performed in triplicate (n = 6). Similar lowercase letters in the error bars indicate insignificant differences (*p* ≥ 0.05) between before and after digestion extracts within the similar assay.

The total phenolic contents (TPC) detected in the undigested extract of Fd-APP was 4.86 ± 0.49 mg GAE/g dw and declined (to 2.91 ± 0.71 mg GAE/g dw and 3.01 ± 0.34 mg GAE/g dw) in the extracts of post-gastric and post-intestinal digestion, respectively. The significant drop (*p* ≤ 0.05) in the TPC after gastric digestion could be attributed to the fact that the degradation of phenolic compounds occurs mainly during gastric digestion [13]. However, the same data (Figure 1) showed insignificant changes (*p* ≥ 0.05) post-intestinal digestion, which confirmed that most polyphenols degradation takes place during gastric digestion. The patterns of changes in the TPC were like those detected in TAC, where data showed significant decrease post-gastric, followed by insignificant (*p* ≥ 0.05) changes in the post-intestinal. The significant decrease in the TPC in the post-gastric could be attributed to the degradation of phenolic compounds during gastric digestion, as reported in the polyphenols of arbutus unedo [13], whole apples [14] and lychee pulp [15]. Furthermore, no significant differences (*p* ≥ 0.05) in the TPC were recorded when comparing post-gastric and post-intestinal results. This observation confirmed the previous suggestion that most PPs degradation occurs during gastric digestion. The antioxidant capacity (AC) is usually determined using various chemical principles, such as the capacity to scavenge free radicals in the form of single electron transfer or hydrogen atom, and chelation of metal ions. Hence, it is not easy to establish the antioxidant potential relying on a single method. The use of various methods is important for detailed evaluation of antioxidant potential in a given food product or pure compounds [16]. Therefore, in the present research, three different methods were applied to evaluate the changes in the antioxidant potential of Fd-APP extracts caused by gastrointestinal digestion. The ABTS, DPPH, and TAC values of Fd-APP extracts during various phases of gastrointestinal digestion are given in Figure 1. All applied methods (ABTS, DPPH and TAC) showed significant (*p* ≤ 0.05) decline in the values of AC after gastric digestion. The ABTS values in undigested extracts (9.69 ± 0.96 mM TE/g dw), decreased significantly (*p* ≤ 0.05) to 5.35 ± 0.67 mM TE/g dw in post-gastric and then increased to 7.33 ± 0.80 mM TE/g dw (*p* ≤ 0.05) post-intestinal digestion. A similar pattern was also observed with DPPH results. Thomas-Valdés et al. [17] attributed the increment in both ABTS and DPPH values of white Chilean strawberries after digestion to the high pH in the intestinal environment (6–7.4). Such conditions can facilitate the degradation of phenolics with enzymes, thus altering their solubility, extractability, and antioxidant activities. Similar findings were also reported by Huang et al. [15], who deduced that the bio accessibility of polyphenols in lychee pulp was the highest under alkaline or neutral environment.

However, the TAC results at post-intestinal were inconsistent with those reported with ABTS and DPPH. Insignificant changes (*p* ≥ 0.05) in the TAC values were reported when comparing post-gastric with post-intestinal results. The recorded amounts of TAC during post-gastric and post-intestinal digestions were 6.99 ± 0.84 and 7.12 ± 0.15 mM TE/g dw, respectively). These unusual fluctuations in the TAC values could possibly be due to the structural variations of certain phenolic compounds after simulated digestion. Li et al. [18] reported a similar degradation of TPC (33.49%), ABTS (27.00%), and DPPH (27.01%) after in vitro digestion of red fleshed apple peel. Bouayed et al. [13] reported that the number of hydroxyl groups associated with aromatic rings in plant cell wall structures may greatly influence antioxidant activity. Moreover, synergistic interactions between polyphenols and other chemical components, such as ascorbic acid and beta-carotene, may contribute to the overall difference in the reported antioxidant activities. This observation is supported by Tsao et al. [19], who reported that different polyphenolic substances exhibit variable antioxidant activities depending on the method of measurement.

### 2.2. Released Individual Polyphenols during In Vitro Gastrointestinal Digestion of Fd-APP

**Figure 2:**
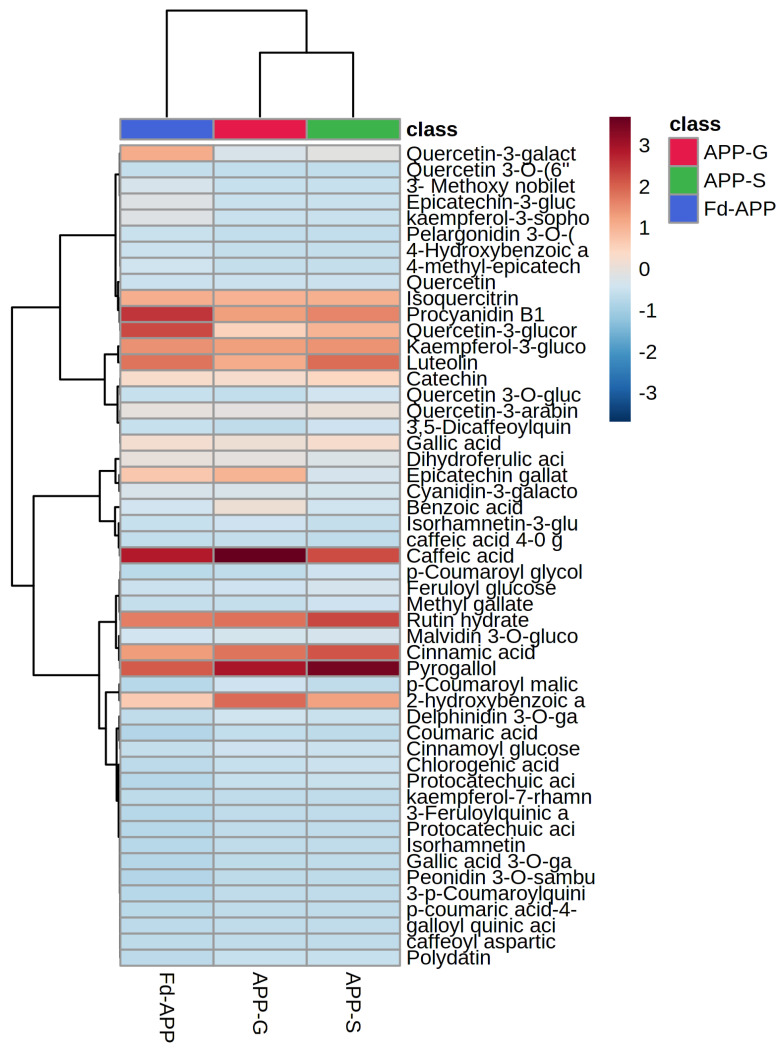
Heatmap showing the concentrations of individual phenolic compounds in Fd-APP (undigested), APP-G (post-gastric) and APP-S (post-intestinal) extracts. Values are expressed as µg/g dw. The full names of the polyphenol compounds present in the heatmap are given in Table S1.

An in vitro digestion model, mimicking the oral, gastric, intestinal, and colonic steps was applied to the Fd-APP to test the stability of individual phenolics during gastrointestinal digestion and fermentation. The phytochemical profile of Fd-APP was not determined at the oral step of in vitro digestion because of minimal modification in the concentration of phenolics, short exposure time (2–3 min), and negligible effects of α-amylase. The exclusion of this step has been justified in many previous studies [13,20]. The simulated gastrointestinal digestion had a significant (*p* ≤ 0.05) impact on the liberation and stability of phenolics in Fd-APP. Data in Figure 2 and Table S1 shows the concentrations of extractable phenolics in undigested, post-gastric, and post-intestinal digestion extracts of Fd-APP. A heatmap was generated to allow thorough visualization of the concentrations of individual phenolics at each stage of in vitro digestion. A total of 51 phenolic compounds from 11 subclasses were quantified in the Fd-APP, and values were expressed as µg/g of apple peel powder on dry weight basis (dw). Results showed that the flavonoids (910.23 ± 63.57 µg/g dw) were highest in concentration representing 57% of total polyphenols, followed by phenolic acids (508.09 ± 50.06 µg/g dw) and anthocyanins (58.77 ± 5.76 µg/g dw) representing 32% and 4%, respectively (Figure 2 and Table S1). Among phenolic acids and flavonoids, hydroxycinnamic acids, flavanols, and flavonols were the most representative compounds. Graziani et al. [20] and Panzella et al. [21] reported higher concentrations of hydroxycinnamates and flavanols in apple peels.

Of the 51 phenolic compounds quantified in Fd-APP before digestion, 34 were detected during the subsequent phases (gastric & intestinal) of digestion. Among these 34 compounds, phenolic acids revealed the highest bio accessibility index (BI) of 67 %, followed by flavonoids (59%), and anthocyanins (32%). To achieve a real balance of the impact of simulated digestion on compounds bio accessibility, a percentage of variation (%var) was calculated for each compound. This %var index provides information about the concentrations of each compound in the soluble fractions of gastric and intestinal digesta in relation to the amounts (µg) quantified per g of Fd-APP prior to digestion. Data in Table S1 showed improved bio accessibility of most phenolic acids during simulated digestion as compared to flavonoids and anthocyanins.

After gastric digestion of Fd-APP, the stability of phenolic acids was slightly affected, hence, the recoveries of benzoic acid and 2-hydroxy benzoic acid (Table S1) were higher (64% and 65.5%, respectively) as compared to the initial concentrations (16.47 ± 2.24 and 53.14 ± 0.45 µg/g dw, respectively). The concentration of gallic acid was also reduced by 28.2%, with a BI of 88%. The compounds that were most heavily affected by gastric digestion included total anthocyanins (−52.5% var), followed by flavonoids (−44.0% var) and stilbenes (−34.1% var). To better understand the phenomena of low recovery, the composition of the food matrix subjected to in vitro digestion is of significant concern. Few studies suggested that the presence of pectin in food matrix may interfere with the release of bioactive compounds into the digestive media due to its intragastric gelation attributes under acidic conditions [22]. Similarly, Mosele et al. [13] and Bouayed et al. [14] reported a minor release of phenolics during gastric incubation of whole apples and Arbutus unedo, respectively. Therefore, varying recovery of phenolics during the gastric phase could be attributed to the differences in withstanding the acidic environments and the web of pectin gel.

Despite the low recovery of some phenolics during gastric phase, amounts were recovered after intestinal digestion. This phenomenon could better be explained by considering the entrapment of some phenolics in pectin gel under acidic conditions (pH 2.5–3.0) of the gastric phase. Whereas the neutral to alkaline conditions (pH 6.5–7.6) during intestinal phase may conveniently disintegrate the gel structure and encourage the release of phenolic compounds [13]. However, the stability of each compound was significantly affected by the time and speed at which the phenolics were liberated from the gel-like assembly of pectin. Higher concentrations of cinnamic acid (101.4 μg/g dw), 2-hydroxybenzoic acid (67.68 ± 10.37 μg/g dw), protocatechuic acid (5.58 ± 0.39 μg/g dw), *p*-coumaroyl glycolic acid (7.50 ± 1.10 μg/g dw), rutin hydrate (105.81 ± 9.37 μg/g dw) and pyrogallol (148.48 ± 20.43 μg/g dw) were recovered after intestinal step as compared to the amounts in undigested and gastric extracts. Concerning the anthocyanin content, a higher variation was observed. The amounts of bio accessible anthocyanins increased at the level of intestinal digestion, with delphinidin 3-*O*-galactoside being highly (*p* ≤ 0.05) bio accessible (67%). In nature, anthocyanins are highly reactive, and tend to degrade easily in foods [23]. Certain factors determine the stability of anthocyanins including medium pH, temperature, their ring substitutes, and attached sugar moieties [24]. Oliveira & Pintado [24] demonstrated highly unstable nature of anthocyanins at slightly alkaline or neutral pH, due to the synthesis of colorless chalcones that consequently disrupt the anthocyanin chromophores. Although the findings of in vitro digestion could not be directly relevant to the in vivo mechanisms, these results could be helpful for food policymakers and future investigations involving the bioactivity and bioavailability of polyphenols in human subjects.

### 2.3. LC-ESI-QTOF-MS/MS Identification of Polyphenols in Fd-APP before and after Simulated Gastrointestinal Digestion

Polyphenols profiles of Fd-APP extracts (before and after digestion) were elucidated using LC-ESI-QTOF-MS/MS analysis (Figure S1). The electrospray mass spectral analysis was performed in negative and positive ionization modes and the results are presented in Table S2. The mass error for molecular ions of all the identified compounds was ≥10.0 ppm. In the present study, we tentatively identified a total of 88 phenolic compounds, with flavonoids being the most frequent class having 35 phenolic compounds including flavonols, flavanone, flavones, flavonols, anthocyanins, dihydrochalcones, dihydroflavanols, isoflavonoids, and eriocitrin. Phenolic acids were the second most abundant class with 27 compounds followed by lignans with 12 compounds. The rest of the identified compounds included hydroxycoumarins (2), hydroxybenzaldehydes (1), hydroxybenzoketones (1), hippuric acid (1), cyclitol (1), furanocoumarins (1), phenolic terpenes (1), esters (1), tyrosols (2), and other polyphenols (3).

#### 2.3.1. Phenolic Acids

As demonstrated in Table S2, the numbers of hydroxybenzoic acids detected in the Fd-APP post gastric (Fd-APP-G) and intestinal (Fd-APP-I) digestion were 3 out of 10 in comparison with undigested samples (Fd-APP). Compound **2** with [M − H]^−^ ions at *m*/*z* 299.0789 was previously detected in the seeds of custard apples [25] and was tentatively characterized as 4-hydroxybenzoic acid 4-*O*-glucoside. The fragmentation yielded product ions at *m*/*z* 255 and *m*/*z* 137, which features the loss of CO_2_ (44 Da) and hexosyl moiety (162 Da), respectively [26]. Compound **6** showed a [M + H]^+^ ion at *m*/*z* 345.0797 in the ESI^−^ MS mode and product ions at *m*/*z* 191, *m*/*z* 169 and *m*/*z* 125 in MS^2^ mode. The first two product ions correspond to the loss of gallic and quinic acid molecules, which yielded *m*/*z* 125 after a loss of CO_2_ moiety (44 Da). This is consistent with fragmentation pattern of galloyl quinic acid, previously identified in the leaves of myrtle [27]. Compound 6 (Figure S2) identified as benzoic acid gave molecular ion at *m*/*z* 121.0295 was detected in Fd-APP only. Compound **10**, tentatively identified as methyl gallate, gave a [M − H]^−^ ion at *m*/*z* 183.0442 that corresponded to the molecular formula C_8_H_8_O_5_, and fragment ions at *m*/*z* 124 [M − H-CO_2_-CH_3_]^−^ and *m*/*z* [M − H-CO_2_-CH_3_-CHO]^−^ [28]. Methyl gallate is the esters derivative of hydroxybenzoic acids particularly gallic acid. Compounds **11** to 26 were tentatively identified as hydroxycinnamic acids and their derivatives (Table S2). Qualitative and quantitative analysis revealed the release of several hydroxycinnamic acids during gastric and/or intestinal phases of simulated digestion. Compound **12** and **17** with pseudo molecular [M + H]^+^ ions at *m*/*z* 281.0669 and *m*/*z* 337.0923, showed MS^2^ fragments at *m*/*z* 235, *m*/*z* 119 and *m*/*z* 265, *m*/*z* 173, *m*/*z* 162 were tentatively identified as *p*-coumaroyl malic acid and 3-*p*-coumaroylquinic acid, respectively. These compounds are derivatives of coumaric acid that are covalently bonded to plant cell walls through various ester linkages and were identified in undigested extract of Fd-APP and after gastric phase of digestion. Compound **14** was identified as cinnamoyl glucose with a pseudo molecular ion [M − H]^−^ at *m*/*z* 309.0992 and yielded product ions at *m*/*z* 147, *m*/*z* 131, *m*/*z* 103 which indicated the loss of hexosyl moiety (162 Da), C_6_H_10_O_6_ (178 Da) and C_7_H_10_O_7_ (206 Da), respectively. It was identified and quantified in all tested sample extracts. Similarly, the analysis of extracts in the TOF-MS mode confirmed the presence of chlorogenic acid *m*/*z* 353.0879 (compound **20**) and feruloyl glucose *m*/*z* 355.1033 (compound **21**) in the before and after digestion extracts of Fd-APP. Compound **26** tentatively identified as *p*-coumaric acid *m*/*z* 163.0415 appeared only in the undigested extracts of Fd-APP. The poor bio accessibility of coumaric acid is associated with its stable nature in digestive environments. However, the release of its esterified compounds during gastric digestion is concomitant with other studies [29,30]. Previous studies have shown limited release of hydroxycinnamic acids from thinned young apples due to their esterified bonding with other food components, along with the pH variations during various phases of simulated digestion. The same authors also demonstrated that the interactions between digestive enzymes and food components also contribute to restrict the bio accessibility of hydroxycinnamic acids [31].

#### 2.3.2. Flavonoids

This class of phenolic compounds is abundant in plants and are the main compounds responsible for free radical scavenging and antioxidant potential in plants [32,33]. In this study 33 flavonoids were identified in Fd-APP extracts before and after digestion. These were further subdivided into flavanols, flavonols, flavones, flavanones, dihydrochalcones, anthocyanins, isoflavonoids, and dihydroflavanols. The identification of compounds **29** (+)-Catechin (*m*/*z* 289.0718) and **28** (-)-epicatechin (*m*/*z* 289.0724) was established using authentic analytical standards. MS/MS fragment ions at *m*/*z* 245 [M − H − 44]^−^ could be attributed to the loss of –CH_2_–CHOH– or CO_2_ moieties, and at *m*/*z* 205 and 179 to the loss of the flavonoid A ring [M − H − 84]^−^ and B ring [M − H − 110]^−^ [34]. The presence of these compounds is widely reported in previous studies of custard apple and fruit pulp [25,34]. Graziani et al. [20] demonstrated that hydroxycinnamic acids, flavanols and procyanidins were the most prominent classes of polyphenols occurring in various apple by-products.

The flavones and flavanones (compounds **34** and **35** in Table S2) were identified as kaempferol-3-glucoside (*m*/*z* 447.0937) and isorhamnetin-3-glucoside (*m*/*z* 477.1065) based on the analytical standards and their MS/MS fragmentation. A previous study reported the release and bio accessibility of these compounds from various parts of apples [20]. Quercetin (compound **37**) *m*/*z* 301.035 was identified in all the methanolic extracts of Fd-APP during each digestive phase. Yet, it demonstrated lower bio accessibility (46%, Table S1). The cardioprotective and anti-inflammatory effects of flavonols have been reported in previous in vitro and animal studies [35,36]. However, the limited bioavailability of pure quercetin reported in human studies was attributed to their rapid metabolism that encourage higher bioavailability of flavonols in their conjugated forms [37]. Rutin, compound **45**, showed molecular ions [M − H]^−^ at *m*/*z* 609.1469 and MS^2^ fragment ions at *m*/*z* 465 and *m*/*z* 301, demonstrated a relatively stable performance during simulated digestion pathway, allowing higher bio accessibility (116%) and detection before and during digestion process. This enhanced concentration of rutin is due to its generation from quercetin being its aglycone form, and the rupture of glycosidic bonds of rhamnose and glucose due to exposure of quercetin to lower gastric pH [38]. Pure rutin from whole asparagus has demonstrated potential in repairing colonic mucosal injury and attenuating the severity of colitis in vivo [39]. Compound **46** showed pseudo molecular ion [M − H]^−^ at *m*/*z* 435.1284 and product ion at *m*/*z* 273 (phloretin- compound **57**) with expected loss of glucoside (162 Da), was tentatively identified as phloridzin- a dihydrochalcone [40]. The detection and bio accessibility of phloridzin in undigested and post gastric extracts in the present study are concomitant with the findings of Tenore et al. [41] in which the authors demonstrated good stability of phloridzin in gastric digestion medium of apple peel and flesh. The same authors reported that exposure of phloridzin to gastric contents may hydrolyse the phenolic glycosides into their aglycone forms during gastric phase of simulated digestion.

Compound **60**, with a [M − H]^−^ at *m*/*z* 303.0501 and MS/MS product ions at *m*/*z* 285 and *m*/*z* 151 was tentatively assigned as taxifolin [23]. The product ions at *m*/*z* 285 corresponded to a loss of H_2_O (18 Da), with the 151-loss attributed to the cleavage of C ring. These compounds were demonstrated to have ample potential in reducing the risk of diabetes, thus are being used for the development of new drugs to control type II diabetes [40]. Anthocyanins are the compounds of plant origin that impart characteristic color to fruits and vegetables and are derivatives of anthocyanidins. A total of 5 anthocyanin compounds (**51**–**55**) were detected in the present research. However, only two of these compounds (**54** and **55**) were identified in raw (undigested) extract of Fd-APP with parent ions at *m*/*z* 532.1269 and *m*/*z* 756.2136, being tentatively identified as pelargonidin 3-*O*-(6′′-succinyl-glucoside) and peonidin 3-*O*-sambubioside-5-*O*-glucoside.

#### 2.3.3. Other Polyphenols

Apart from phenolic acids and flavonoids, various other compounds from following subclasses were identified, including: hydroxycoumarins, hydroxybenzaldehyde, hydroxybenzoketones, hippuric acid, cyslitol, eriocitrin, furanocoumarins, phenolic terpenes, esters, tyrosols, stilbenes, and lignans. Compounds **63** and **64** with [M − H]^−^ and [M + H]^+^ molecular ions at *m*/*z* 243.0302 and 163.0405 were identified in undigested extracts only (Table S2). Similarly, norathyriol (compound **66**), vanilloylglycine (compound **67**), hydroxytyrosol 4-*O*-glucoside (compound **72**), phlorin (compound **74**), salvianolic acid B (compound **75**) and *d*-viniferin (compound **88**) were detected in undigested fractions only. Some of the compounds from the above subclasses were released only during various stages of in vitro digestion. The release of piceatannol (compound **86**) was monitored after gastric and intestinal steps only, with no detection in undigested extract. It showed precursor ion at *m*/*z* 243.0677 and gave fragment ions at *m*/*z* 225, 201 and 159, which were generated from the expected loss of water molecule and successive losses of C_2_H_2_O [42]. Stilbenes are reported to perform various bioactive functions such as resistance against bacterial and fungal infections and improve cardiovascular functionalities [43]. With regards to lignans, compounds **77** (7-oxomatairesinol [M + H]^+^ ions at *m*/*z* 373.1302) and **78** (matairesinol [M + H]^+^ ions at *m*/*z* 359.1469) were detected in the extracts of Fd-APP. Previously, compound **78** (matairesinol) was characterized in various genotypes of custard apple by-products [25]. Compound **79** (Secoisolariciresinol) with precursor ion at *m*/*z* 363.179 was detected in the gastric and intestinal fractions of Fd-APP in negative and positive ion modes and designated as secoisolariciresinol. The release profile of various lignan compounds showed high variations. As is evident from Table S2, compounds 80 to 84 were detected only after the intestinal digestion of Fd-APP. These varied release patterns of lignans may correspond to the hydrolysis of compounds by the action of enzymes and variations in pH [17]. The compound piceatannol (number 86) was detected after gastric and intestinal steps only, with no detection in undigested extract. It showed a precursor ion at *m*/*z* 243.0677 and gave fragment ions at *m*/*z* 225, 201 and 159, which were generated from the expected loss of water molecule and successive losses of C_2_H_2_O.

### 2.4. Inhibition of α-glucosidase and α-amylase Activities

**Figure 3:**
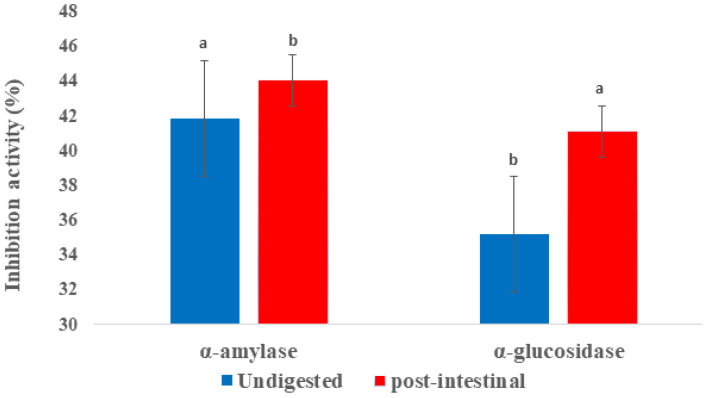
α-amylase and α-glucosidase inhibition (%) of phenolics released from Fd-APP before and after in vitro gastrointestinal digestion. The values are means of tests performed in triplicate (n = 6). Similar lowercase letters in the error bars indicate insignificant differences (*p* ≥ 0.05) between before and after digestion extracts within similar assays.

α-amylase is one of the key enzymes that are responsible for absorption of glucose and hydrolysis of starch into oligosaccharides and disaccharides. The function of α-glucosidase is the hydrolysis of these into monosaccharides to facilitate absorption, thus increasing glucose levels in the blood. Therefore, the inhibition of these enzymes is required to alleviate the symptoms of type 2 diabetes. Data in Figure 3 presented the α-amylase and α-glucosidase inhibitory activities of Fd-APP phenolic extracts before and after in vitro digestion. The inhibitory action against α-amylase increased significantly (*p* ≤ 0.05) from 41.88% (undigested) to 44.08% (post-intestinal digestion). Similarly, the phenolic extracts of Fd-APP exhibited significantly higher (*p* ≤ 0.05) α-glucosidase inhibition (approximately 6% increase) in the post-digestion extracts. The higher inhibitory potential in extracts post-intestinal digestion indicated no correlation with the results of total phenolic content, which implies that the higher inhibition rate is more pertinent to the specific type of polyphenols (ring structures) than total phenolic concentration [44]. Diverse phenolic compounds in the extracts could act synergistically or antagonistically with these saccharide hydrolyzing enzymes and may alter their inhibition potential. The substantial variations in the structures of different phenolic groups determine their capability to bind digestive enzymes, hence the distinct phenolic composition in the analyzed extracts could have dictated this outcome. Previous studies by Spínola et al. [44] and Wojdyło et al. [45] also reported poor correlations between total phenolic contents of *Elaeagnus umbeletta* and cherry fruit extracts and the inhibition effects against α-glucosidase and α-amylase activities.

### 2.5. Production of Short Chain Fatty Acids (SCFAs) during In Vitro Colonic Fermentation of Fd-APP

**Figure 4:**
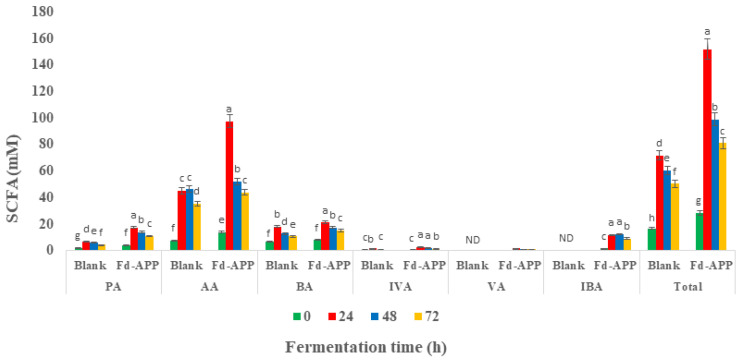
Concentration of SCFAs (mM) in Fd-APP and blank during 72 h of fecal fermentation. Different letters in the same comparison block indicate significant differences (*p* ≤ 0.05). Note: PA—propionic acid; AA—acetic acid; BA—butyric acid; IVA—isovaleric acid; VA—valeric acid; IBA—isobutyric acid; total—total SCFAs.

The concentrations of the SCFAs throughout the different fermentation/incubation timepoints (0–72 h) are shown in Figure 4 and Table S3. The shifts and significant (*p* ≤ 0.05) increments observed in the various SCFAs contents along the fermentation timepoints agreed with the pH variations, which will be discussed later (Figure 6B). However, changes in SCFAs were marked higher in the cultures supplemented with carbon source (Fd-APP) than in the blank cultures. The trends of individual SCFAs were similar as the total tendency, since their production reached the peak at 24 h of fermentation both in Fd-APP supplied media and blank (fecal materials only), except for iso-butyric acid that reached maximum (12.22 ± 1.33 mM) after 48 h of fermentation. Among the detected SCFAs, acetic acid (AA) was the most predominant SCFA, that reached a highest concentration of 97.53 ± 9.09 mM in the medium supplied with non-digestible fraction of Fd-APP. This prevalent formation of acetic acid is consistent with the findings of Koutsos et al. [3] who investigated fecal fermentation of some commercial apple varieties. AA is the main end-product of carbohydrate fermentation by bifidobacteria, also being formed by the action of anaerobic genera from the human gut microflora [46]. Besides acetic acid, propionic and butyric were the major acids (major short chain fatty acids) produced in larger concentrations ranging from 4 mM to 24 mM, while iso-butyric, iso-valeric, valeric and heptanoic acids (minor short chain fatty acids) produced at minute concentration ranging from 1 to 12 mM. These results are concomitant to the findings of Koutsos et al. [3] and Tamargo et al. [47] who reported increased production of acetic, propionic, and butyric acids during the in vitro fermentation of some commercial apple varieties and cranberry extracts.

Butyrate production is of great relevance to the gut epithelium, as it alters the propagation of epithelial cells, and its enhanced production by intestinal flora has been associated with inhibition and/or lower rate of colon cancer [48]. Propionate is primarily used by the liver and converted into glucose. Hosseini et al. [49] suggested a prospective role of propionate in modulating the synthesis of cholesterol. Data in Table S3 showed significant differences (*p* ≤ 0.05) in the levels of detected major and minor short chain fatty acids. The minor SCFAs are reported to induce apoptosis in breast cancer cells and could have a regulatory impact on anti-inflammatory cytokine expression [50]. It is also worth mentioning here that the generation of SCFAs in the media lacking a carbon source (blank) was due to the degradation of proteins by putrefactive gut microbiota.

### 2.6. Changes in Phenolic Compounds during Colonic Fermentation of Indigestible Fraction of Fd-APP

**Figure 5:**
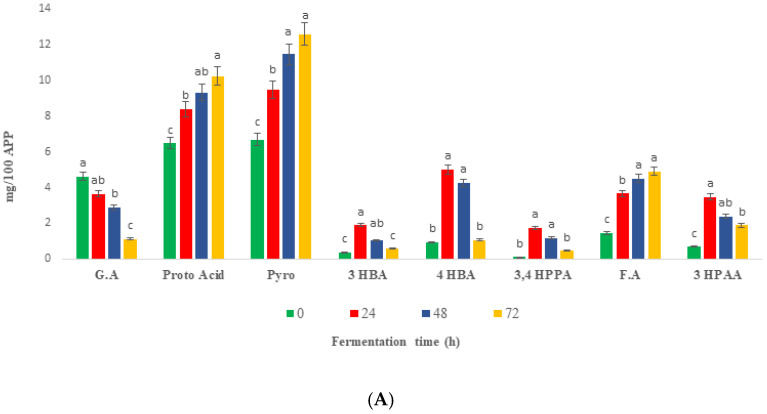

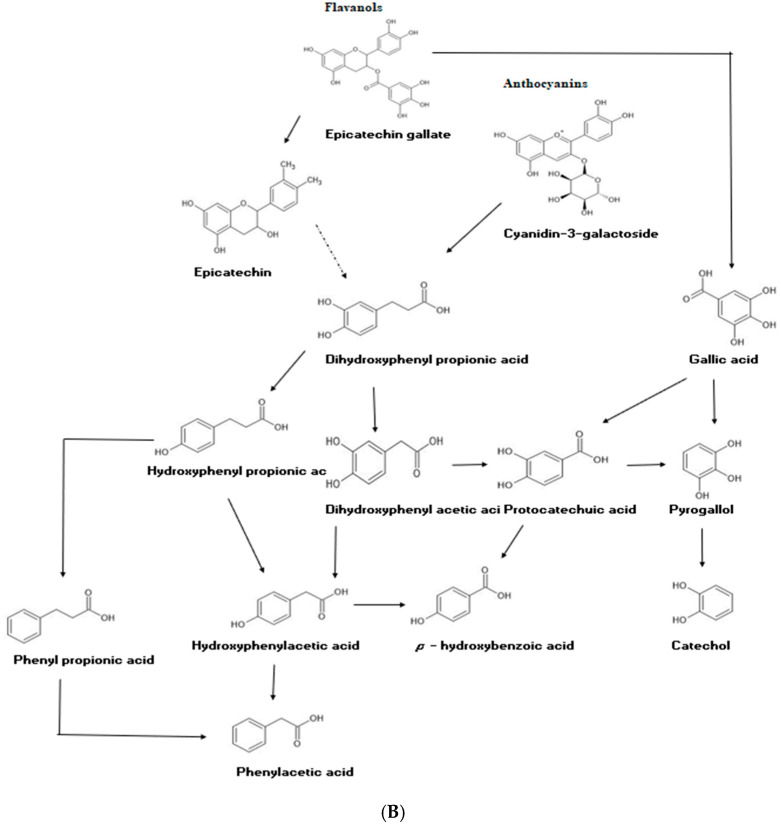
(**A**)—Phenolic metabolites of Fd-APP produced during in vitro colonic fermentation of Fd-APP. G.A—gallic acid, Proto. A—Protocatechuic acid, 3,4 HPPA—3,4 hydroxyphenyl propionic acid, 3 HPPA—3 hydroxyphenyl acetic acid, Pyro—pyrogallol, 4 HBA; 4 hydroxybenzoic acid, 3 HBA: 3 hydroxybenzoic acid, F.A: ferulic acid. Different letters within the same metabolic compound indicate significant differences (*p* ≤ 0.05) among various fermentation timepoints. The results are expressed as mg/100 g APP. (**B**)—Proposed metabolic pathways of polyphenols from Fd-APP by gut microbiota.

The major phenolic compounds and their catabolism during 72 h of fermentation are shown in Figure 5. Due to the varying intensities of phenolic compounds under different ionization conditions of LC-ESI-QTOF-MS/MS, quantification of fecal catabolites was preferred using HPLC-PDA for complex fecal solutions. Inoculating and incubating the insoluble fraction of Fd-APP with feces extract revealed significantly (*p* ≤ 0.05) different trends of phenolic compounds than those incubated without feces (blank). Therefore, the phenolic compounds detected in both Fd-APP residue plus fecal and blanks were ignored. Only the parent compounds specific to the action of microbiota were studied. The intact parent phenolic acids and flavonoids were majorly catabolized in the first 24 h of fermentation, while the concentration of most produced metabolites decreased gradually during 48–72 h of fermentation with some exceptions. Protocatechuic acid (PA), pyrogallol and ferulic acid (FA) showed different responses to fermentation and continued to increase in quantities till 72 h of fermentation (Figure 5A). The following detected metabolite; 3- hydroxyphenyl acetic acid (3-HPAA), ferulic acid (F.A), 3-(4-hydroxyphenyl) propionic acid (3,4 HPPA) and 4- hydroxybenzoic acid (4-HBA) were previously reported as metabolites of human fecal fermentation of polyphenols in date seed powder [51]. Previous studies investigating the microbial metabolism of dietary catechins reported the production of benzoic, phenyl lactic, phenyl propanoic, phenyl acetic, and phenyl valeric acids derivatives by various hydroxylation patterns [52], however, valerolactones and phenyl valeric acids were not detected in the present study. The progressive catabolism (75%) of gallic acid (GA) during 72 h of fecal fermentation could be attributed to the consumption of GA as a substrate for maintenance of bacterial fermentation and/or conversion into PA by dihydroxylation reactions [53]. The accumulation and formation of PA during colonic fermentation is consistent with the previous proposed conversion of GA to PA. Jiménez-Girón et al. [54] indicated that generation of 4 hydroxybenzoic acid (4 HBA) and PA by colonic fermentation of flavanols, involves a series of catabolic reactions such as C-ring fission, decarboxylation, dihydroxylation, and oxidation reactions (Figure 5B).

The other phenolic compounds detected in fecal fermented media were FA and pyrogallol. FA is usually found associated with arabinoxylans in the plants cell walls, therefore, a higher proportion of it can be released via the action of the microbial enzymes on plants cell walls [51,55]. Higher concentration of pyrogallol along the fecal fermentation could be attributed to the reciprocal interactions between fecal LAB and phenolic compounds. Results from previous studies revealed the consumption of GA by various strains of lacticaseibacilli and its subsequent degradation into pyrogallol, following decarboxylation pathways [56,57].

### 2.7. Changes in Bacterial Population and pH during Fecal Fermentation

**Figure 6:**
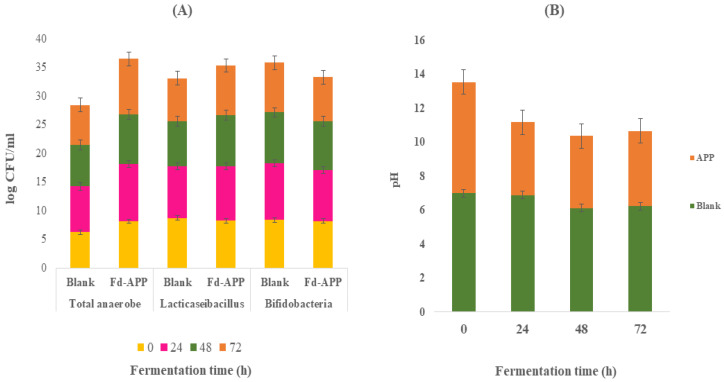
Variations in microbial count (**A**) and pH (**B**) during in vitro colonic fermentation of Fd-APP.

The quantitative changes in the human fecal bacterial populations observed during the in vitro fermentation are given in (Figure 6). Blank cultures (no added Fd-APP) showed insignificant (*p* ≥ 0.05) changes in the total anaerobic, *Lacticaseibacillus* or *Bifidobacteria* counts throughout the 72 h of fermentation. However, fecal samples with added Fd-APP substrate revealed significant (*p* ≤ 0.05) increases in total anaerobic, Lactobacillus and *Bifidobacteria* counts after 48 and 72 h of fermentation (Figure 6A). For example, data showed that *Bifidobacterium* counts increased by 1.90 log CFU/mL after 24 h of fermentation. These results were supported by a previous study which suggested that foods rich in polyphenols, such as Fd-APP, might have a certain influence upon gut microbial activity and composition by preventing or promoting the growth of particular bacterial groups [58]. Such changes in microbial population and composition may also cause catabolic reactions of phenolics, that facilitate the release of 3,4 HPPA, 4HBA and 3PAA following decarboxylation and/or reduction in phenolic acids as discussed before. The pH values of Fd-APP/fecal mix declined significantly (*p* ≤ 0.05) after 24 h of fermentation (Figure 6B) when compared with the blank (fecal only). On the contrary, blanks showed no changes in pH values during fermentation. Furthermore, the reported decline in the pH after 24 h of samples (Fd-APP/fecal mix) fermentation remained stable throughout the fermentation periods (72 h). Such a decline in the pH value in the presence of Fd-APP could be attributed to the fermentation of Fd-APP and the release of some SCFAs as discussed previously (Section 3.5).

## 3. Materials and Methods

### 3.1. Chemicals and Reagents

Caffeic acid, quercetin, quercetin-3-galactoside, gallic acid, *p*-coumaric acid, chlorogenic acid, rutin, luteolin, phloretin, epicatechin, kaempferol-3-glucoside, catechin, quercetin, procyanidin B1, pyrogallol, epicatechin 3′-O-gallate, 3-hydroxybenzoic acid, protocatechuic acid, benzoic acid, cinnamic acid, polydatin, Folin-Ciocalteu phenol reagent (FCR), α-amylase from *aspergillus oryzae*, porcine pepsin, acarbose, rat intestinal powder, ammonium molybdate, Trolox (6-hydroxy-2,5,7,8-tetramethylchroman-2-carboxylic acid), DPPH (2,2-diphenyl-1-picrylhydrazyl), ABTS 2,2′-azino-bis (3-ethylbenzothiazoline-6-sulfonic acid), *p*-nitrophenyl-α-d-glucopyranoside, potassium persulfate, bile salts, LC-MS grade formic acid, and HPLC grade acetic acid were purchased from Sigma-Aldrich (Castle Hill, NSW, Australia). Pancreatin was obtained from Alfa Aesar (Ward Hill, MA, USA). NaCl, Na_2_CO_3_, KCl, NaOH, HCl, NaHCO_3_, (NH_4_)_2_CO_3_, MgCl_2_(H_2_O)_6_, CaCl_2_, KH_2_PO_4_, K_2_HPO_4_, Na_3_PO_4,_ HPLC grade methanol and acetonitrile were purchased from Chem-supply Pty Ltd. (Melbourne, VIC, Australia).

### 3.2. Preparation of Sample and Digestion Fluids

Freeze-dried apple peel powder (Fd-APP) was prepared as described by Zahid et al. [59] using ripened pink lady cultivar. The stock digestion salivary fluid (SF), gastric fluid (GF), and intestinal fluid (IF) were prepared following the method of [60]. Details about the composition of digestion fluids are given in our previously published manuscript [61].

### 3.3. In Vitro Sequential Digestion of Fd-APP

The prepared Fd-APP samples were subjected to in vitro digestion based on the modified protocols of Minekus et al. [60]. The salivary mastication was achieved by mixing 3 g of powdered sample with 6 mL SF and the pH was adjusted to 7.2 ± 0.1, followed by addition of 75 U mL^−1^ α-amylase and 0.3 M CaCl_2_. The whole mixture was incubated at 37 °C for 2–4 min, under constant agitation. Afterwards, simulated GF along with 0.3 M CaCl_2_ and freshly prepared 2000 U mL^−1^ pepsin were added at 1:1 *v*/*v*, and the pH was adjusted to 3.0 ± 0.2 with sterile 6 M HCl, and the digesta was incubated at 37 °C for 2 h in a shaking incubator (ZWYR-240, Labwit, Ashwood, VIC, Australia). Thereafter, an aliquot (5 mL) was separated as gastric digesta. The remaining mixture was corrected to pH 7.0 ± 0.1 using a sterile solution of 1 M NaOH and intestinal phase was proceeded by adding IF (1:1 *v*/*v*) including 100 U mL^−1^ pancreatin, and 10 mM bile salts (10 mM), followed by incubation at 37 °C for 2 h with sporadic shaking, and collection of aliquots (5 mL) at the end of small intestinal digestion. Soluble and insoluble fractions from each collected aliquots were separated by centrifugation (5500× *g*, 4 °C for 15 min) and the separated fractions were immediately frozen in liquid nitrogen and stored at −20 °C until further analyses.

### 3.4. In Vitro Colonic Fermentation

An ethical approval (ID: 1954660.1) was obtained from the Ethics Advisory Group in the Faculty of Veterinary and Agricultural Sciences, The University of Melbourne, before starting this in vitro study. Immediately after the simulated digestion, the insoluble fraction was submitted to colonic fermentation under anaerobiosis by adopting the modified method of Sirisena et al. [51]. A 6.9 ± 1.0 pH bacterial fermentation medium (BFM) was prepared and sterilized (20 min, 121 °C). The BFM medium was prepared by dissolving Guar (1.0 g), bile salts (0.4 g), casein (3 g), CaCl_2_ (0.11 g), KCl (4.5 g), KH_2_PO_4_ (0.5 g), K_2_HPO_4_ (0.5 g), Cysteine HCl (0.8 g), mucin (4 g), MgSO_4_.7H_2_O (1.23 g), NaCl (4.5 g), NaHCO_3_ (1.5 g), pectin (2 g), peptone (5 g), potato starch (5 g), tryptone (5 g), yeast extract (4.5 g), and Tween 80 (1.0 mL) in 1000 mL Milli-Q water using a volumetric flask.

Freshly voided fecal samples were collected from two healthy donors (aged between 30–33 years). The donors declared no probiotic and antibiotic consumption during the last 3 months and had no gastric diseases by the time of collection. The feces were transported on ice, pooled, and homogenized with 0.1 M sterilized phosphate buffer (pH 7.0) using a stomacher mixer. The mixture was then filtered through a bilayer cheesecloth to acquire a single fecal slurry at a ratio of 20:80 (*w*/*w*) feces: buffer. Aliquots (5 mL) of fecal slurry were dispensed into 50 mL N_2_ flushed corning tubes and the fermentation was proceeded after adding 0.5 g pellets (insoluble intestinal fraction) and 5 mL of the prepared BFM. The tubes (triplicates) were tightly closed, sealed, and incubated in shaking incubator (ZWTR-240, Labwit, China) under anaerobic conditions (37 °C, 120 rpm). Anaerobiosis was generated using anaerobic gas generator (AN 0010W, Oxoid^®^) in anaerobic chambers (BD BBL^™^ Gas Pak^™^, Australia). The blank test was conducted by inoculating FS (5 mL) and BFM at 1:1 ratio, without sample to correct for reagent contribution. The sample tubes were collected and analyzed at various fermentation time points (0, 24, 48 and 72 h). The fermentation was stopped after 72 h by placing the tubes in an ice bath. Samples were collected at each fermentation time to monitor changes in pH and microbial counts. The supernatant fraction from each tube was separated by centrifugation (10,000× *g*, 15 min, 4 °C), and stored at −80 °C until analysis of phenolic metabolites, phytochemical contents, and short chain fatty acids production.

### 3.5. Extraction and Determination of Polyphenols

The total phenolic content (TPC) was determined using Folin-Ciocalteu reagent, and antioxidant capacities (AC) were analyzed applying ABTS, DPPH, TAC methods following the modified methods of Peng et al. [62] and Zahid et al. [61,63]. A proper volume of each sample extract was mixed with a relevant reagent (Folin-Ciocalteu, ABTS, DPPH, and phosphomolybdenum dye), and optical density was measured at specific wavelengths using 96-well microplate reader (Multiskan Go, Thermo Fisher Scientific, Vantaa, Finland) against the standard curves of gallic acid (0-200 µg/mL) for TPC and Trolox (0–100 mM/mL) for AC.

### 3.6. Evaluation of Phenolic Profile by HPLC-DAD-ESI-QTOF-MS/MS

The separation and quantification of phenolic compounds in Fd-APP before and after simulated in vitro digestion was performed through a Waters 2690 Alliance Separation Module (Waters, Rydalmere NSW, Australia) coupled with a Waters 2998 Photodiode Array Detector (PDA). Sample extracts (20 µL) were injected onto a C18 Synergi hydro-RP column (4.6 × 250 mm, particle size of 4μm) protected by a C18 ODS guard column (4.0 × 3.0 mm) (Phenomenex, Lane cove, NSW, Australia). The LC operation conditions were column compartment temperature (25 °C), and PDA detection between 190–520 nm with the monitoring wavelengths of 280 nm, 320, and 370 nm. The binary phase consisted of 0.1% formic acid in water (eluent A) and acetonitrile (eluent B). The flow rate was 800 µL/min with a 60 min gradient elution program for B: 0 min (10% B), 10 min (20% B), 15 min (30% B), 20 min (40% B), 25 min (50% B), 30 min (60% B), 40 min (80% B), 45 min (90% B), 50 min (100% B), 55 min (100% B), 58 min (10% B), 60 min (5% B). MS analysis was performed using Agilent 6520 Accurate Mass LC-QTOF-MS/MS (Agilent Technologies, Santa Clara, CA, USA) for identification of phenolics. The LC-QTOF-MS/MS analysis was based on the modified and optimized methods of Zahid et al. [61] and Ali et al. [26]. The same column and operation conditions were used for HPLC analysis except for injection volume and flow rate, which were 6 µL and 0.6 mL/min, respectively. The mass spectra were acquired both in negative and positive ionization modes using electrospray ionization (ESI) in the range of 100–1000 *m*/*z*. The ESI was set to operate under following conditions; drying gas flow rate was 9 mL/min at 325 °C, nebulizer pressure of 45 psi, collision gas was nitrogen, collision energies were 10, 20, 40- eV, and capillary and nozzle voltage were 3500 V and 500 V, respectively.

#### Data Interpretation

The data were analyzed using Agilent LC/MS Mass Hunter Qualitative Software (Agilent Technologies, Santa Clara, CA, USA), Personal Compounds Database and Library (PCDL) for metabolites, and FooDB (www.foodb.ca, accessed on 28 August 2022) and PubChem (https://pubchem.ncbi.nlm.nih.gov/, accessed on 28 August 2022). A total of 25 authentic standards were used for generation of equations through LC MS/MS, of which 20 compounds from the analyzed samples matched the analytical standards. Out of the total 80 identified phenolic compounds some were semi-quantified without standards and based on subclass and structural similarities such as functional groups and core structure. Such methods of analysis and semi-quantitation have been applied in previous studies [23,26].

### 3.7. Analysis of α-Glucosidase and α-Amylase Inhibitory Activities

The determination of α-glucosidase and α-amylase inhibitory activities of phenolic extracts before and after in vitro digestion were performed following the methods of Xiong et al. [64]. The phenolic extracts (25 µL each) were mixed with 90 µL potassium phosphate buffer-A (0.12 M, pH 6.8), 25 µL α-glucosidase enzyme solution from rat intestinal acetone powder and 25 µL of *p*-nitrophenyl-α-d-glucopyranoside solution (pNGP, 25 mM) in each cell of 96-well microplate. The mixture was incubated at 37 °C for 65 min, and the absorbance was measured at 405 nm using plate reader (Thermo Fisher Scientific, Waltham, MA, USA). Acarbose was used as a positive control at a concentration of 0.5 mg/mL. The inhibition was calculated using the following formula:

(1) $$\mathrm{Inhibition}\;(\%)=\left[1-\left(\frac{A\;sample-A\;sample\;background}{A\;control-A\;control\;background}\right)\right]\times 100$$

where *A_sample_* represents absorbance of test sample, *A_sample background_* represents absorbance with pNGP swapped by solvent, *A_control_* is absorbance with the sample replaced by buffer, *A_control background_* represents sample and pNGP replaced by buffer and solvent, respectively.

Similarly, α-amylase inhibition was assessed using porcine pancreas α-amylase at 250 units/mL in sodium phosphate buffer-B (20 mM, 7.0 mM NaCl,1 mM CaCl2, pH 6.8) as a working solution. A soluble starch solution of 1% was prepared in sodium phosphate buffer-C (20 mM, 7.0 mM NaCl, pH 6.8). The reaction mixtures (20 µL sample extract, 90 µL sodium phosphate buffer-C and 60 µL working solution of α-amylase) were mixed in the wells of a 96-well microplate. Thereafter, the reaction was initiated by adding 50 µL of the 1% soluble starch. The 96-well plate was incubated for 50 min at 37 °C. The plate was then wrapped in aluminum foil before cooling the contents in an ice water bath. This step was followed by adding 100 µL dimethyl sulfoxide to each well to dissolve precipitates and the absorbance was measured at 405 nm using microplate reader (Multiskan Go, Thermo Fisher Scientific, Vantaa, Finland). The inhibitory activity was determined using formula 1.

### 3.8. Analysis of SCFAs by GC-FID

The concentration of SCFAs in the colonic digesta was evaluated using the modified protocol of Gu et al. [65]. Supernatants (1.5 mL) obtained after colonic fermentation were mixed via vertexing with four volumes of internal standard mixture containing 1% formic acid, 1% orthophosphoric acid and 4-methyl valeric acid, internal standard solution at a concentration of 1.59 mmol/L. A specific volume (1 mL) of each sample mixture was dispensed into 1.5 mL Eppendorf tubes and centrifuged at 10,000× *g* for 10 min at 4 °C. The supernatant was collected and stored at 4 °C until the GC analysis was performed. Acetic, propionic, butyric, valeric, iso-valeric, iso-butyric and heptanoic acids relative to the internal standard were used as analytical standards to construct standard curves. Sample and standard volumes of 2 µL were injected into gas chromatograph (7890B Agilent, CA, USA), equipped with a capillary column (SGE BP21, 12 × 0.53 mm internal diameter with 0.5 µm film thickness, SGE International, Ringwood, VIC, Australia, P/N 054473), a flame ionization detector (FID), an autosampler (Gilson GX-271, Gilson Inc., Middleton, WI, USA) and autoinjector. The temperatures set for FID and injection port were 240 and 200 °C, respectively. Helium was used as a carrier gas at a flow rate of 14.4 mL/min. Nitrogen, hydrogen and air were used as makeup gases.

### 3.9. Microbiological Analysis and pH

The quantitative variations in fecal microbiota were assessed using a conventional method of spread plate employing plate count agar (PCA), MRS agar and MRS agar enriched with cysteine to examine total anaerobic count, *Lacticaseibacillus* and *Bifidobacterium* counts, respectively [66]. The initial enumeration of the blank was carried out with fecal slurry (5 mL) mixed with sterile basal medium (5 mL) followed by serial dilutions using 0.1% buffered peptone water, spread plated, and incubation was performed at 37 °C for 48 h. Similar procedures were repeated with the tested samples, where the in vitro fecal fermentation was carried out using the insoluble part (residue) from intestinal digesta. For testing, samples (1 mL) were aseptically collected at various time points (0 h, 24 h, 48 h and 72 h) during fermentation and were subjected to pH monitoring and microbial analysis.

### 3.10. Statistical Analysis

All quantitative measurements involved three replicate samples with at least two measurements within each sample. Data were collected, subjected to one way analysis of variance (ANOVA) followed by Tukey′s post hoc test to separate the means. The statistical tests were run with Minitab statistical package (version 20.0.) and MetaboAnalyst 5.0 (https://www.metaboanalyst.ca/, accessed on 1 September 2022). The data was presented as mean values ± standard deviation.

## 4. Conclusions

This study investigated the effect of in vitro digestion of Fd-APP on the release of phenolic compound and antioxidant activities and their indirect effect on hypoglycemic inhibition activities. The study also evaluated the effect of pre-digested polyphenols on the production and release of short chain fatty acids (SCFA), phenolic metabolites and acidification parameters during fecal fermentation. Our results indicated that Fd-APP showed moderate recovery of polyphenols during in vitro digestion, however the released phenolics demonstrated antioxidant and glycemic inhibitory activities. Meanwhile, the bound polyphenols in the residual fractions were metabolized during colonic fermentation, yielding various phenolic catabolites in parallel with the generation of SCFA. The in vitro colonic fermentation of Fd-APP residues caused significant release of phenolic compounds that could possibly support gut health by their prebiotic like functions. The investigation proposes some significant understandings into the potential benefits of apple peel polyphenols on the health of gastrointestinal tract. Nevertheless, the health promoting effects of phenolic metabolites from apple peel via in vivo have not been fully investigated. Consequently, additional research is recommended to evaluate the in vivo healthful effects of Fd-APP and the primary mechanisms.

## Data Availability

The data presented in this study are available on request from the corresponding author. The data will be made publicly available upon publication.

## Supplementary Materials

The following supporting information can be downloaded at: https://www.mdpi.com/article/10.3390/ijms24021514/s1.
