# Supplementary material for: Identification of Phenolics Profile in Freeze-Dried Apple Peel and Their Bioactivities during In Vitro Digestion and Colonic Fermentation
Source: Int J Mol Sci. 2023 Jan 12;24(2):1514. doi: 10.3390/ijms24021514 (PMC9864335; doi:10.3390/ijms24021514)
Supplement: Supplementary file 1 [file ijms-24-01514-s001.zip › ijms-2118422-supplementary.pdf]

Supplementary materials

Table S1: Apple peel polyphenols bioaccessibility during simulated gastrointestinal digestion (μg/g dw)

Phenolic compounds	Undigested	Gastric stage	Var (%)	Intestinal stage	Var (%)	BI (%)
**Phenolic acids**						
Gallic acid	37.75±7.64[a]	27.10±4.48[b]	-28.2	33.27±5.35[a]	-11.9	88
Caffeic acid	132.34±12.16[b]	148.94±8.38[a]	12.5	104.30±12.51[c]	-21.2	79
Chlorogenic acid	5.71±0.63[a]	4.26±0.11[ab]	-25.4	6.22±0.21[a]	8.9	74
Coumaric acid	2.11±0.09[a]	2.13±0.5[a]	0.95	Nd	Nd	Nd
Benzoic acid	16.47±2.24[b]	27.01±0.09[a]	64.0	8.68±1.78c	-47.3	52
Cinnamic acid	77.44±10.77[b]	83.98±11.12[b]	8.4	101.40±10.62[a]	30.9	148
2-hydroxybenzoic acid	53.14±0.45[c]	87.94±14.89[a]	65.5	67.68±10.37[b]	27.4	84
Protocatechuic acid	3.78±0.22[bc]	4.40±0.35[ab]	16.4	5.58±0.39[a]	47.6	93
p-Coumaroyl malic acid	4.17±0.22[b]	7.48±0.55[a]	13.4	Nd	Nd	Nd
3-p-Coumaroylquinic acid	4.39±0.37[a]	Nd	Nd	Nd	Nd	Nd
p-Coumaric acid-4-*O*-glucoside	5.25±0.56[a]	Nd	Nd	Nd	Nd	Nd
Caffeoyl aspartic acid	7.50±0.14[a]	Nd	Nd	Nd	Nd	Nd
Caffeic acid 4-*O* glucoside	8.95±2.01[a]	3.21±0.28[b]	-31.2	Nd	Nd	Nd
3,5-Dicaffeoylquinic acid	10.98±1.57[a]	6.77±0.46[b]	-14.8	Nd	Nd	Nd
p-Coumaroyl glycolic acid	5.63±0.32[b]	Nd	Nd	7.50±1.10[a]	33.2	133
Feruloyl glucose	12.84±2.51[a]	7.26±1.11[b]	-43.5	11.37±2.24[a]	-8.8	57

3-Feruloylquinic acid	4.02±1.22[a]	Nd	Nd	Nd	Nd	Nd
Cinnamoyl glucose	10.08±0.76[a]	6.80±0.55[b]	-14.7	6.27±0.59[b]	-37.8	62
Methyl gallate	9.73±0.11[a]	3.38±0.18[c]	-65.3	7.44±0.79[b]	-23.5	76
Gallic acid 3-*O*-gallate	2.78±0.13[a]	Nd	Nd	Nd	Nd	Nd
4-Hydroxybenzoic acid 4-*O*-glucoside	13.53±1.99[a]	Nd	Nd	Nd	Nd	Nd
Protocatechuic acid 4-*O*-glucoside	3.61±0.08[a]	Nd	Nd	Nd	Nd	Nd
Galloyl quinic acid	5.48±0.06[a]	Nd	Nd	Nd	Nd	Nd
Dihydroferulic acid-4-sulfate	30.41±3.81[a]	20.74±1.54[b]	-31.8	15.91±1.11[c]	-47.7	52
**Total phenolic acids**	508.09±50.06[a]	441.40±44.59[b]	-13.1	375.62±47.06[c]	-26.1	68
**Flavonoids**						
Quercetin	13.32±0.30[a]	5.78±1.65[b]	-34.1	6.07±2.31[a]	5.6	46
Isorhamnetin	3.75±0.29[a]	Nd	Nd	Nd	Nd	Nd
Epicatechin gallate	55.79±0.91[a]	56.19±0.33[a]	0.7	Nd	Nd	Nd
Kaempferol-3-glucoside	82.57±5.49[a]	66.24±5.63[c]	-19.8	74.39±4.29[b]	-9.9	80
Procyanidin B1	121.44±15.65[a]	66.17±8.99[c]	-45.5	80.16±12.76[b]	-34.0	66
Luteolin	94.90±2.97[a]	60.69±1.55[b]	-36.0	91.13±4.03[a]	-4.0	64
Rutin hydrate	91.06±4.32b	84.93±0.36[c]	-6.7	105.81±9.37[a]	16.2	116
Quercetin-3-glucoronide	112.9±12.42[a]	40.76±4.65[c]	-63.9	59.03±7.25[b]	-47.7	36
Quercetin-3-galactoside	69.99±9.26[a]	12.93±0.40[c]	-81.5	19.13±0.90[b]	-72.7	18
Isoquercitrin	69.56±0.84[a]	57.21±0.31[b]	-17.8	60.55±0.79[a]	-13.0	83

Catechin	41.56±0.66[a]	31.72±1.71[b]	-23.7	38.76±0.33[a]	-6.7	76
Epicatechin-3-glucuronide	24.13±1.91[a]	Nd	Nd	Nd	Nd	Nd
4-methyl-epicatechin-3-glucuronide	15.49±0.38[a]	Nd	Nd	Nd	Nd	Nd
Quercetin-3-arabinoside	30.14±1.97[a]	19.47±2.38[c]	-35.4	25.70±4.77[b]	-14.7	85
3- Methoxy nobiletin	19.31±2.73[a]	Nd	Nd	Nd	Nd	Nd
Quercetin 3-*O*-(6"-malonyl)-glucoside	10.59±0.32[a]	Nd	Nd	Nd	Nd	Nd
Quercetin 3-*O*-glucosyl-xyloside	10.83±0.41[a]	Nd	Nd	9.41±0.42[a]	-13.1	87
kaempferol-3-sophoroside-7-rhamnoside	24.46±1.84[a]	Nd	Nd	Nd	Nd	Nd
kaempferol-7-rhamnoside	6.85±0.25[a]	Nd	Nd	Nd	Nd	Nd
Isorhamnetin-3-glucoside	11.59±0.70[a]	7.48±0.44[b]	-35.5	3.32±0.42[c]	-71.4	28
**Total flavonoids**	910.23±63.57[a]	509.57±32.45[b]	-44.0	573.46±47.64[c]	-37.0	63
**Anthocyanins**						
Cyanidin-3-galactoside	20.66±1.52[a]	13.28±2.02[b]	-35.7	10.42±2.17[b]	-49.6	50
Malvidin 3-*O*-glucoside	15.88±2.81[a]	9.74±0.58	-38.7	11.64±0.19[b]	-26.7	61
Delphinidin 3-*O*-galactoside	8.19±0.67[a]	7.77±0.64[b]	-31.3	5.62±0.37[c]	-29.5	67
Peonidin 3-*O*-sambubioside-5-*O*-glucoside	1.74±0.17[a]	Nd		Nd	Nd	Nd
Pelargonidin 3-*O*-(6"-succinyl-glucoside)	12.30±0.59[a]	Nd	Nd	Nd	Nd	Nd
**Total anthocyanins**	58.77±5.76[a]	27.93±3.39[b]	-52.5	30.54±2.58[c]	-48.0	55

**Stilbenes**						
Polydatin	5.54±0.31[a]	3.65±0.29[ab]	-34.1	4.16±0.34[a]	-24.9	75
**Other polyphenols**						
Pyrogallol	105.44±7.63[c]	124.19±19.46[b]	17.8	148.48±20.43[a]	40.8	141

The results are expressed as µg/g of Fd-APP submitted to digestion. Similar superscript letters within the same row represent non-significant differences ($p \geq 0.05$).

Nd: not detected

% Var: concentration of each compound (µg) present in the digesta after gastric or duodenal digestions in relation to the amount quantified per g of Fd-APP submitted to *in vitro* digestion.

Table S2: Identification of phenolic compounds using LC-ESI-QTOF-MS^2 in undigested, gastric, and intestinal fractions of Fd-APP

No	Proposed compounds	Molecular Formula	RT (min)	Ionization (ESI +/-)	Molecular Weight	Theoretical (m/z)	Observed (m/z)	Mass Error (ppm)	MS/MS product ions	Samples***
**Phenolic acids**										
**Hydroxybenzoic acids**										
**1**	*Gallic acid	$C_7H_6O_5$	7.727	[M-H]-	170.1202	169.0142	169.0144	1.2	125	Fd-APP, Fd-APP-G, Fd-APP-I
**2**	4-Hydroxybenzoic acid 4- *O* glucoside	$C_{13}H_{16}O_8$	9.907	[M-H]-	300.0845	299.0772	299.0789	5.6842	255, 137	Fd-APP
**3**	* Protocatechuic acid	$C_7H_6O_4$	12.287	[M-H]-	154.1201	153.0193	153.0195	1.3	109, 91	APP, APP-G, APP-I
**4**	Protocatechuic acid 4-*O*-glucoside	$C_{13}H_{16}O_9$	9.322	[M-H]-	316.0794	315.0721	315.0744	7.2999	153	Fd-APP
**5**	Gallic acid 3-*O*-gallate	$C_{14}H_{10}O_9$	15.498	[M-H]-	322.0325	321.0252	321.0245	-2.1805	303, 275, 169	Fd-APP
**6**	Galloyl quinic acid	$C_{14}H_{16}O_{10}$	5.941	$[M+H]^+$	344.0744	345.0817	345.0797	-5.7957	169, 125	Fd-APP
**7**	*Benzoic acid	$C_7H_6O_2$	21.019	[M-H]-	122.0368	121.0295	121.0286	-7.4362	103, 77	Fd-APP
**8**	3-Hydroxybenzoic acid	$C_7H_6O_3$	6.242	[M-H]-	138.0317	137.0244	137.0267	6.7853	115, 93	Fd-APP
**9**	2-Hydroxybenzoic acid	$C_7H_6O_3$	4.3	[M-H]-	138.0317	137.0244	137.0257	9.4874	93, 65	Fd-APP
**10**	Methyl gallate	$C_8H_8O_5$	3.61	[M-H]-	184.0372	183.0445	183.0442	-1.6212	124, 95	Fd-APP, Fd-APP-G, Fd-APP-I
**Hydroxycinnamic acids**										
**11**	p-Coumaroyl glycolic acid	$C_{11}H_{10}O_5$	4.003	$[M+H]^+$	222.0528	223.0601	223.0594	-3.1382	147, 119	Fd-APP
**12**	p-Coumaroyl malic acid	$C_{13}H_{12}O_7$	4.974	$[M+H]^+$	280.0583	281.0656	281.0669	4.6253	235, 119	Fd-APP-G, Fd-APP-I
**13**	Caffeoyl aspartic acid	$C_{13}H_{13}NO_7$	3.852	$[M+H]^+$	295.0692	296.0765	296.0783	6.0795	278, 132	Fd-APP
**14**	Cinnamoyl glucose	$C_{15}H_{18}O_7$	32.204	[M-H]-	310.1052	309.0979	309.0992	4.2058	147, 131, 103	Fd-APP, Fd-APP-G, Fd-APP-I
**15**	p-Coumaric acid 4-*O*-glucoside	$C_{15}H_{18}O_8$	14.06	[M-H]-	326.1002	325.0929	325.0926	-0.9228	163	Fd-APP
**16**	Caffeic acid 4-*O*-glucoside	$C_{15}H_{18}O_9$	14.151	[M-H]-	342.0951	341.0878	341.0889	3.2245	179, 161	Fd-APP
**17**	3-p-Coumaroylquinic acid	$C_{16}H_{18}O_8$	18.183	[M-H]-	338.1002	337.0929	337.0923	-1.7799	265, 173, 162	Fd-APP, Fd-APP-G
**18**	* Cinnamic acid	$C_9H_8O_2$	8.802	[M-H]-	148.1586	147.0451	147.0447	-2.7	103	Fd-APP, Fd-APP-G, Fd-APP-I
**19**	*Caffeic acid	$C_9H_8O_4$	16.69	[M-H]-	180.1631	179.035	179.0341	-5.0	161, 135	Fd-APP, Fd-APP-G, Fd-APP-I
**20**	*Chlorogenic acid	$C_{16}H_{18}O_9$	4.302	[M-H]-	354.0951	353.0878	353.0879	0.2832	253, 190, 144	Fd-APP, Fd-APP-G, Fd-APP-I
**21**	Feruloyl glucose	$C_{16}H_{20}O_9$	17.564	[M-H]-	356.1107	355.1034	355.1033	-0.2816	339, 177	Fd-APP, Fd-APP-G, Fd-APP-I

	**22**	3-Feruloylquinic acid	$C_{17}H_{20}O_9$	58.02	$[M+H]^+$**	368.1107	369.118	369.1169	-2.9800	351, 163, 149	Fd-APP
	**23**	3,5-Dicaffeoylquinic acid	$C_{25}H_{24}O_{12}$	3.485	[M-H]-	516.1268	515.1195	515.1249	10.4830	353, 191, 179	Fd-APP-G
	**24**	Verbascoside	$C_{29}H_{36}O_{15}$	4.335	[M-H]-	624.2054	623.1981	623.2034	8.5045	161	Fd-APP
	**25**	2,5-di-S-Glutathionyl caftaric acid	$C_{33}H_{42}N_6O_{21}S_2$	3.852	$[M+H]^+$	922.1844	923.1917	923.1918	0.1083	774, 689, 547	Fd-APP
	**26**	*p-Coumaric acid	$C_9H_8O_3$	14.06	$[M-H]_-$	164.0473	163.04	163.0415	9.2002	145, 119	Fd-APP
**Hydroxyphenylpropanoic acids**											
	**27**	Dihydroferulic acid 4-sulfate	$C_{10}H_{12}O_7S$	4.487	[M-H]-	276.0304	275.0231	275.0235	1.4544	230	Fd-APP, Fd-APP-G, Fd-APP-I
**Flavonoids**											
**Flavanols**											
	**28**	*Epicatechin	$C_{15}H_{14}O_6$	17.682	[M-H]-**	290.079	289.0717	289.0724	2.4215	137, 125	Fd-APP, Fd-APP-G, Fd-APP-I
	**29**	*(+)-Catechin	$C_{15}H_{14}O_6$	24.83	[M-H]-**	290.079	289.0717	289.0718	0.3459	245, 205, 179	Fd-APP, Fd-APP-G, Fd-APP-I
	**30**	(-)-Epigallocatechin 3'-*O*-glucuronide	$C_{21}H_{22}O_{13}$	15.498	[M-H]-	482.106	481.0987	481.0991	0.8314	153, 321	Fd-APP
	**31**	* (-)-Epicatechin 3-*O*-gallate	$C_{22}H_{18}O_{10}$	18.25	[M-H]-	442.2132	441.0827	441.0832	1.1	289, 271, 169, 125	Fd-APP, Fd-APP-G
	**32**	4'-*O*-Methyl-(-)-epigallocatechin 3'-*O*-glucuronide	$C_{22}H_{24}O_{13}$	3.677	[M-H]-	496.1217	495.1144	495.1124	-4.0395	149, 121	Fd-APP
	**33**	*Procyanidin dimer B1	$C_{30}H_{26}O_{12}$	4.34	[M-H]-	578.1424	577.1351	577.1377	4.5050	451	Fd-APP, Fd-APP-G, Fd-APP-I
**Flavones**											
	**34**	kaempferol-7-rhamnoside	$C_{21}H_{20}O_{10}$	29.493	[M-H]-	432.1056	431.0983	431.0979	-0.9279	285, 151	Fd-APP
	**35**	*Kaempferol-3-glucoside	$C_{21}H_{20}O_{11}$	25.425	[M-H]-**	448.1006	447.0933	447.0937	0.8947	284, 255	Fd-APP, Fd-APP-G, Fd-APP-I
**Flavanones**											
	**36**	Isorhamnetin-3-glucoside	$C_{22}H_{22}O_{12}$	4.725	[M-H]-	478.1111	477.1038	477.1065	5.6591	301, 175, 113, 85	Fd-APP, Fd-APP-G, Fd-APP-I
	**37**	Narirutin	$C_{27}H_{32}O_{14}$	24.736	[M-H]-	580.1792	579.1719	579.1749	5.1798	434, 418	Fd-APP
**Flavonols**											
	**38**	*Quercetin	$C_{15}H_{10}O_7$	4.688	[M-H]-**	302.0426	301.0353	301.035	-0.9966	127, 302, 285	Fd-APP, Fd-APP-G, Fd-APP-I
	**39**	Isorhamnetin	$C_{16}H_{12}O_7$	26.033	[M+H] +	316.0583	317.0656	317.0631	-7.8848	302, 153	Fd-APP
	**40**	Quercetin 3-*O*-arabinoside	$C_{20}H_{18}O_{11}$	4.834	[M-H]-**	434.0849	433.0776	433.0792	3.6945	303	Fd-APP-G, Fd-APP-I
	**41**	Quercetin 3'-*O*-glucuronide	$C_{21}H_{18}O_{13}$	26.12	[M-H]-	478.0747	477.0674	477.0683	1.8865	301	Fd-APP
	**42**	*Quercetin-3-galactoside	$C_{21}H_{20}O_{12}$	23.576	[M-H]-**	464.0955	463.0882	463.0873	-1.9435	301	Fd-APP-G, Fd-APP-I
	**43**	3-Methoxynobiletin	$C_{22}H_{24}O_9$	13.03	[M-H]-	432.142	431.1347	431.1371	5.5667	373, 161	Fd-APP

	**44**	Quercetin 3-*O*-(6''-malonyl)-glucoside	$C_{24}H_{22}O_{15}$	25.55	[M-H]-	550.0959	549.0886	549.0902	2.9139	503, 151	Fd-APP
	**45**	Quercetin 3-*O*-glucosyl-xyloside	$C_{26}H_{28}O_{16}$	4.343	[M-H]-	596.1377	595.1304	595.1295	-1.5123	303, 287, 597	Fd-APP, Fd-APP-I
	**46**	*Rutin	$C_{27}H_{30}O_{16}$	4.084	[M-H]-	610.1534	609.1461	609.1469	1.3133	465, 301	Fd-APP, Fd-APP-G, Fd-APP-I
**Dihydrochalcones**											
	**47**	Phloridzin	$C_{21}H_{24}O_{10}$	27.849	[M-H]-	436.137	435.1297	435.1284	-2.9876	273	Fd-APP, Fd-APP-G
	**48**	3-Hydroxyphloretin 2'-*O*-glucoside	$C_{21}H_{24}O_{11}$	24.83	[M-H]-	452.1319	451.1246	451.1225	-4.655	289, 273	Fd-APP
	**49**	Phloretin 2'-*O*-xylosyl-glucoside	$C_{26}H_{32}O_{14}$	24.91	[M-H]-	568.1792	567.1719	567.1696	-4.0552	549, 435, 273	Fd-APP
	**50**	3-Hydroxyphloretin 2'-*O*-xylosyl-glucoside	$C_{26}H_{32}O_{15}$	22.227	[M-H]-	584.1741	583.1668	583.1646	-3.7725	525, 289, 140	Fd-APP
**Anthocyanins**											
	**51**	*Cyanidin 3-*O*-galactoside	$C_{21}H_{21}O_{11}$	4.768	[M-H]-	449.1084	448.1011	448.0984	-6.0254	284, 255, 211	Fd-APP-G, Fd-APP-I
	**52**	Delphinidin 3-*O*-galactoside	$C_{21}H_{21}O_{12}$	4.725	[M-H]-	465.1033	464.096	464.0961	0.2155	303	Fd-APP-G, Fd-APP-I
	**53**	Malvidin 3-*O*-glucoside	$C_{23}H_{25}O_{12}$	52.172	[M-H]-	493.1346	492.1273	492.1272	-0.2032	179, 161	Fd-APP, Fd-APP-I
	**54**	Pelargonidin 3-*O*-(6''-succinyl-glucoside)	$C_{25}H_{25}O_{13}$	55.932	[M-H]-	533.1295	532.1222	532.1269	8.8326	429, 285, 117	Fd-APP
	**55**	Peonidin 3-*O*-sambubioside-5-*O*-glucoside	$C_{33}H_{41}O_{20}$	4.085	[M-H]-	757.2191	756.2118	756.2136	2.3803	626, 463	Fd-APP
**Isoflavonoids**											
	**56**	*Luteolin	$C_{15}H_{10}O_{6}$	15.193	[M+H]+	286.0477	287.055	287.0565	5.2255	285, 201	Fd-APP, Fd-APP-G, Fd-APP-I
	**57**	xanthoxyletin	$C_{15}H_{14}O_{4}$	4.117	[M-H]-	258.0892	257.0819	257.0832	5.0568	215, 185	Fd-APP-G
	**58**	*Phloretin	$C_{15}H_{14}O_{5}$	27.849	[M-H]-	274.0841	273.0768	273.0756	-4.3944	167	Fd-APP, Fd-APP-G
	**59**	6''-*O*-Acetylglycitin	$C_{24}H_{24}O_{11}$	4.085	[M-H]-	488.1319	487.1246	487.129	9.0326	283, 267	Fd-APP
**Dihydroflavanols**											
	**60**	Taxifolin	$C_{15}H_{12}O_{7}$	4.117	[M-H]-	304.0583	303.051	303.0481	-9.5693	285, 151	Fd-APP-G
	**61**	Dihydromyricetin 3-*O*-rhamnoside	$C_{21}H_{22}O_{12}$	4.725	[M-H]-	466.1111	465.1038	465.1026	-2.5801	353, 313, 151	Fd-APP-G
**Eriocitrin**											
	**62**	Eriocitrin	$C_{27}H_{32}O_{15}$	4.085	[M-H]-	596.1741	595.1668	595.169	3.6964	459, 287, 286	Fd-APP
**Hydroxycoumarins**											
	**63**	Urolithin C	$C_{13}H_{8}O_{5}$	23.545	[M-H]-	244.0372	243.0299	243.0302	1.2344	215, 198, 187, 169	Fd-APP
	**64**	4-Hydroxycoumarin	$C_{9}H_{6}O_{3}$	14.841	[M+H]+	162.0317	163.039	163.0405	9.2001	121	Fd-APP
**Hydroxybenzaldehydes**											

	**65**	p-Anisaldehyde	$C_8H_8O_2$	4.945	[M-H]-	136.0524	135.0451	135.0462	8.1454	122, 109, 94	Fd-APP-G
**Hydroxybenzoketones**											
	**66**	Norathyriol	$C_{13}H_8O_6$	3.714	[M-H]-	260.0321	259.0248	259.0228	-7.7213	241, 231, 189, 109	Fd-APP
**Hippuric acid**											
	**67**	Vanilloylglycine	$C_{10}H_{11}NO_5$	4.495	[M+H]+	225.0637	226.071	226.0707	-1.3270	164, 107	Fd-APP
**Cyslitol**											
	**68**	Quinic Acid	$C_7H_{12}O_6$	4.004	[M-H]-	192.0634	191.0561	191.0573	6.2809	173, 127, 85	Fd-APP, Fd-APP-G, Fd-APP-I
**Furanocoumarins**											
	**69**	5-Methoxyfuranocoumarin	$C_{12}H_8O_4$	3.965	[M-H]-	216.0423	215.035	215.0348	-0.9301	200, 172	Fd-APP, Fd-APP-G, Fd-APP-I
**Phenolic terpenes**											
	**70**	Carvacrol	$C_{10}H_{14}O$	57.308	[M-H]-	150.1045	149.0972	149.0978	4.0242	132, 108	Fd-APP-G, Fd-APP-I
**Esters**											
	**71**	24-Methylcholestanol ferulate	$C_{38}H_{58}O_4$	57.066	[M+H]+	578.4335	579.4408	579.4435	4.6597	533, 441	Fd-APP-G
**Tyrosols**											
	**72**	Hydroxytyrosol 4-*O*-glucoside	$C_{14}H_{20}O_8$	17.045	[M+H]+	316.1158	317.1231	317.1217	-4.4147	285, 135	Fd-APP
	**73**	p-HPEA-EDA	$C_{17}H_{20}O_5$	59.173	[M+H]+	304.1311	305.1384	305.1406	7.2098	291, 259	Fd-APP-I
**Other polyphenols**											
	**74**	Phlorin	$C_{12}H_{16}O_8$	4.003	[M+H]+	288.0845	289.0918	289.0898	-6.9182	272, 237, 179	Fd-APP
	**75**	Salvianolic acid B	$C_{36}H_{30}O_{16}$	4.17	[M-H]-	718.1534	717.1461	717.1455	-0.8366	699, 537	Fd-APP
	**76**	*Pyrogallol	$C_6H_6O_3$	4.128	[M-H]-**	126.0317	125.0244	125.0253	7.1986	97, 81	Fd-APP, Fd-APP-G, Fd-APP-I
**Lignans**											
	**77**	7-Oxomatairesinol	$C_{20}H_{20}O_7$	58.553	[M+H] +	372.1209	373.1282	373.1302	5.3601	355, 249	Fd-APP
	**78**	Matairesinol	$C_{20}H_{22}O_6$	32.094	[M+H]+	358.1416	359.1489	359.1469	-5.5687	341, 137	Fd-APP
	**79**	Secoisolariciresinol	$C_{20}H_{26}O_6$	4.029	[M+H]+**	362.1729	363.1802	363.179	-3.3041	165, 121	Fd-APP-G, Fd-APP-I
	**80**	Syringaresinol	$C_{22}H_{26}O_8$	3.879	[M+H]+	418.1628	419.1701	419.1743	10.0198	415, 345	Fd-APP-I
	**81**	Schisandrin B	$C_{23}H_{28}O_6$	3.161	[M+H]+	400.1886	401.1959	401.1967	1.9940	299, 226	Fd-APP-I
	**82**	Schisanhenol	$C_{23}H_{30}O_6$	58.459	[M+H]+	402.2042	403.2115	403.2104	-2.7281	385, 271	Fd-APP-I

	**83**	Deoxyschisandrin	$C_{24}H_{32}O_6$	4.029	[M+H]+	416.2199	417.2272	417.2278	1.4381	402, 347, 361, 301	Fd-APP-I
	**84**	Schisandrin	$C_{24}H_{32}O_7$	54.847	[M-H]-	432.2148	431.2075	431.2091	3.7105	415, 301	Fd-APP-I
	**85**	Tigloylgomicin H	$C_{28}H_{36}O_8$	23.321	[M-H]-	500.241	499.2337	499.2349	2.4037	483, 401	Fd-APP
**Stilbenes**											
	**86**	Piceatannol	$C_{14}H_{12}O_4$	4.035	ESI - / [M-H]-	244.0736	243.0663	243.0677	5.7597	225, 201, 159	Fd-APP-G, Fd-APP-I
	**87**	*Polydatin	$C_{20}H_{22}O_8$	3.147	ESI - / [M-H]-	390.1315	389.1242	389.123	-3.0838	227, 185	Fd-APP, Fd-APP-G, Fd-APP-I
	**88**	d-Viniferin	$C_{28}H_{22}O_6$	19.127	ESI - / [M-H]-	454.1416	453.1343	453.1347	0.8827	451, 347, 289, 93	Fd-APP

*Represent the detection of compounds using authentic standards.

**Denotes the detection of compounds in both positive $[M + H]^+$ and negative $[M – H]^-$ ionization modes.

***Samples were designated as following: Fd-APP (undigested), Fd-APP-G (post gastric) and Fd-APP-I (post intestinal digestions).

Table S3: Concentrations of individual and total SCFAs in Fd-APP and control (blank) fermentation products

**Samples**	**Time (h)**	**SCFAs (mM)**						
		Acetic acid	Propionic acid	Butyric acid	Iso-butyric acid	Valeric acid	Iso-valeric acid	Total SCFAs
Fd-APP	0	13.72±0.38^{e}	4.05±0.61^{f}	7.96±1.42^{f}	1.67±0.28^{c}	0.14±0.02^{c}	0.76±0.09^{c}	28.30±1.20^{g}
	24	97.53±9.09^{a}	17.47±0.29^{a}	21.04±0.70^{a}	11.63±1.03^{a}	1.50±0.01^{a}	2.61±0.15^{a}	151.77±11.98^{a}
	48	52.06±0.38^{b}	13.96±0.22^{b}	17.46±0.13^{b}	12.22±1.33^{a}	1.06±0.06^{b}	2.12±0.05^{a}	98.89±0.95^{b}
	72	43.71±0.76^{c}	10.96±0.77^{c}	15.06±1.77^{c}	9.04±1.31^{b}	0.98±0.06^{b}	1.13±0.36^{b}	80.88±1.70^{c}
Blank	0	7.44±1.64^{f}	2.11±0.01^{g}	6.55±0.73^{f}	Nd	Nd	0.57±0.09^{c}	16.67±1.99^{h}
	24	45.05±0.36^{c}	6.83±0.69^{d}	18.13±2.24^{b}	Nd	Nd	1.67±0.21^{b}	71.68±2.30^{d}
	48	41.24±1.82^{c}	5.75±0.47^{e}	12.68±1.03^{d}	Nd	Nd	0.58±0.20^{c}	60.25±2.16^{e}
	72	35.35±0.91^{d}	4.31±0.57^{f}	10.61±1.77^{e}	Nd	Nd	Nd	50.27±1.39^{f}

Values are presented as means ± standard deviation. Values with similar letters within the same column are not significantly ($p \geq 0.05$) different. Nd: not detected

(A)

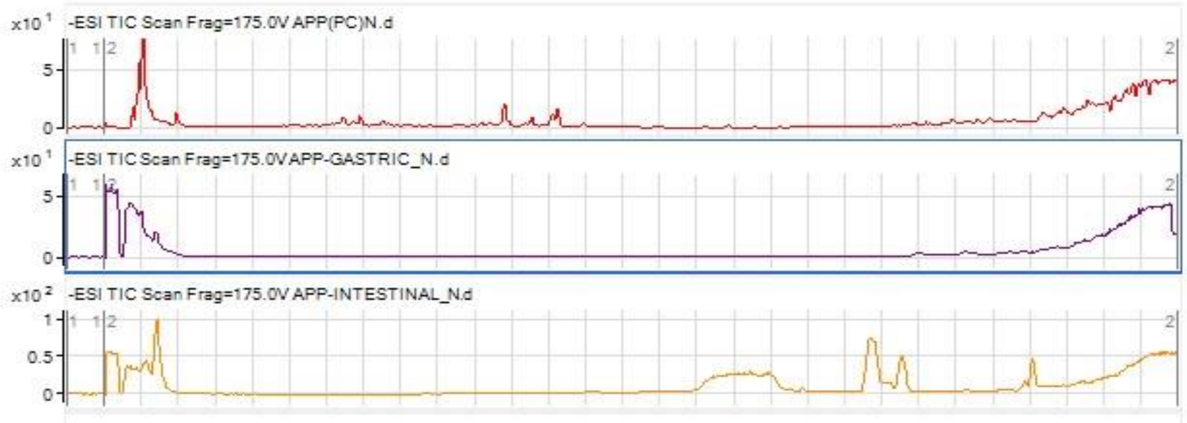

(B)

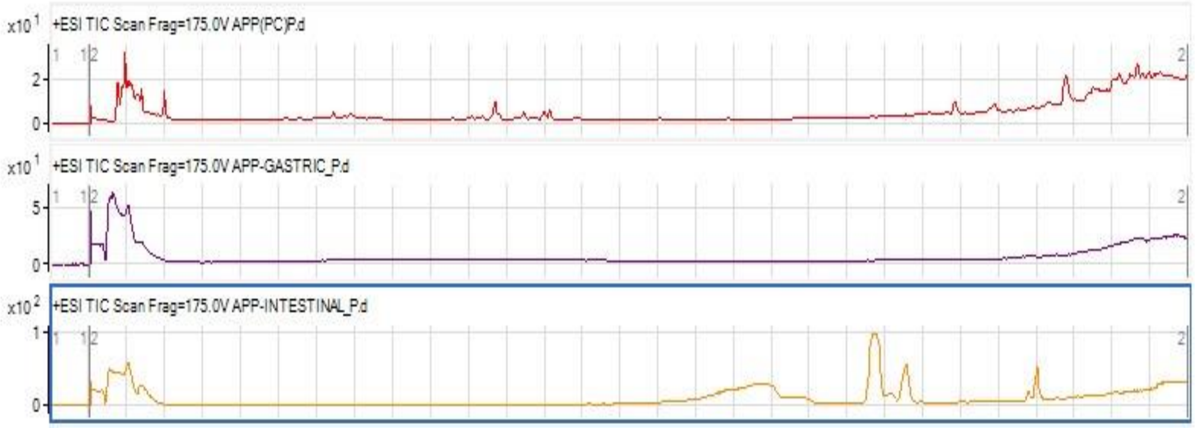

Figure S1: Representative total ion chromatograms (TIC) of Fd-APP identified in negative (A) and positive (B) ionization modes *via* LC-QTOF-MS/MS.

(A)

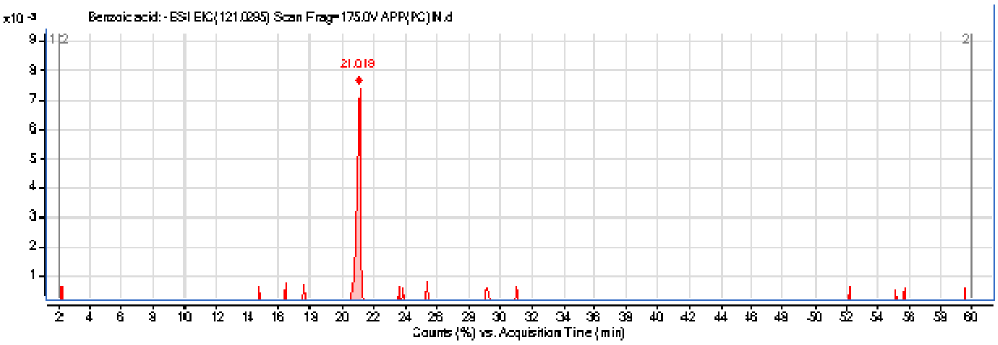

(B)

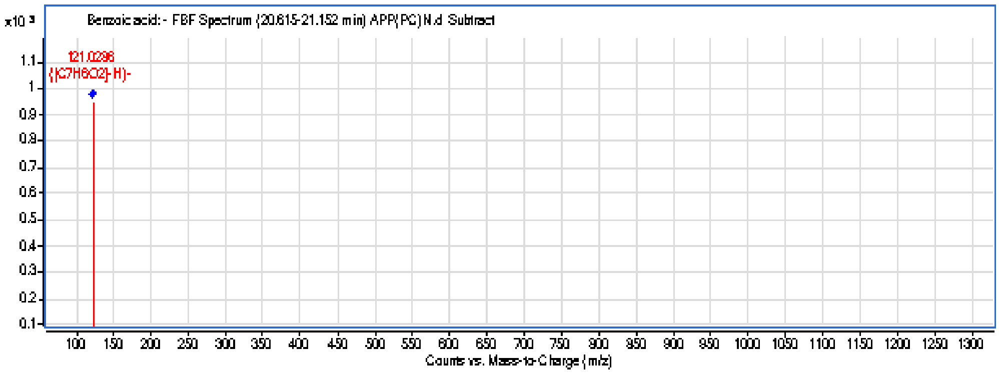

Figure S2: Chromatogram (A) and mass spectrum (B) of benzoic acid (compound 6) identified in raw extracts of Fd-APP.
